# Renal Mitochondrial Lipid Peroxidation during Sepsis

**DOI:** 10.4172/jok.1000116

**Published:** 2016-02-29

**Authors:** P Singh, N Parajuli, PR Mayeux, LA MacMillan-Crow

**Affiliations:** Department of Pharmacology and Toxicology, University of Arkansas for Medical Sciences, AR, USA

**Keywords:** Sepsis, Kidney, Mitochondria, Lipid peroxidation

## Abstract

Sepsis can provoke kidney injury, which increases mortality. Human and animal studies have documented increased renal oxidative injury and mitochondrial damage during sepsis. However, few studies have attempted to dissect specific renal targets and/or types of oxidative injury using the cecal ligation and puncture (CLP) murine model of sepsis. The purpose of this short communication is to examine the extent of lipid peroxidation within renal mitochondria using CLP and blue native gel electrophoresis which separates intact mitochondrial respiratory complexes. Our results show that CLP induced increased 4-hydroxy-nonenal protein adduction (marker of lipid peroxidation) in renal homogenates and mitochondrial fractions. Blue native gel electrophoresis revealed that respiratory complex III was selectively targeted within mitochondrial fractions. This supports our prior report showing renal complex III inactivation following CLP. Future studies will identify specific renal proteins within complex III that are modified during sepsis to provide mechanistic insight on how mitochondrial respiration is inhibited during sepsis.

## Introduction

Sepsis is the 9^th^ leading cause of death in patients 65 to 75 years of age. It also accounts for ~50% of acute kidney injury (AKI) cases, and the onset of AKI in septic patients increases mortality to as high as 70% [1]. The renal epithelium is uniquely rich in mitochondria due to the high-energy demand of transport processes. It is well established that oxidative injury and mitochondrial damage are pivotal events during sepsis-mediated damage [2–9]. Takasu et al. analyzed renal biopsy samples from human nonsurvivors of sepsis and showed an increase in mitochondrial injury compared to kidneys of nonseptic patients [10]. Recently using the clinically relevant cecal ligation and puncture (CLP) murine model of polymicrobial sepsis, we showed that delivery of a mitochondrial targeted antioxidant (MitoTEMPO) reduced mitochondrial damage and tyrosine nitration of renal proteins (as a marker of oxidative stress) [9]. These data suggest that mitochondrial oxidants play a detrimental role in renal mitochondrial function during sepsis. However, the mechanism of oxidant-induced injury to the mitochondria during sepsis is unknown.

Peroxidation of membrane lipids leads to fragmentation of polyunsaturated fatty acids resulting in the production of various cytotoxic and highly reactive aldehydes such as 4-hydroxy-2-nonenal (4-HNE) [11]. 4-HNE is a highly reactive compound, which can modify cysteine, histidine, and lysine residues within proteins and also DNA [12]. Exogenously added 4-HNE has also been shown to inhibit mitochondrial function [13,14]. Similar to other reactive oxygen species such as peroxynitrite and hydrogen peroxide, 4-HNE can lead to dose-dependent biological effects. Low levels have been shown to be involved with physiological signaling, while excessive levels can produce toxicity/damage [15–17].

The goal of this short communication is to identify whether lipid peroxidation within the mitochondria is increased within the kidney during sepsis using the CLP murine model of sepsis. To assess lipid peroxidation, we measured 4-HNE using a 4-HNE antibody in renal homogenates and mitochondrial fractions following induction of sepsis by CLP.

## Materials and Methods

### Cecal ligation and puncture (CLP) murine model of sepsis

CLP was performed in male 40-week-old C57/BL6 mice (The Jackson Laboratory, Bar Harbor, ME), as described previously [6,9]. The cecum was ligated 1.5 cm from the tip with a 4-0 silk suture and punctured twice with a 21 gauge needle. The cecum was isolated but neither ligated nor punctured in control sham-operated mice (Sham). All mice received buprenorphine for analgesia at the time of surgery and antibiotics at 6 h. Animals were housed and handled in accordance to *National Institute of Health Guide for the Care of Laboratory Animals* with approval by the Institutional Animal Care and Use Committee at the University of Arkansas for Medical Sciences.

### Western blot analysis

Renal extracts (50 μg) from kidney homogenates were resolved onto SDS-PAGE gel and then transferred to PVDF membrane. Western blot analysis was performed using antibodies against 4-HNE (Abcam, #ab46545; 500) and β-actin (Sigma, #A5441; 1:1000). Densitometry evaluations on scanned membranes were performed using AlphaEase FC software.

### Blue native polyacrylamide gel electrophoresis (BN-PAGE)

Mitochondria isolation from kidneys of Sham and CLP groups was performed using sucrose containing buffer as described previously [18,19]. Mitochondrial complexes were extracted from the isolated mitochondria (250 μg) using 10% *n*-dodecyl-β-D-maltoside and 0.5 M aminocaproic acid (detergent/protein ratio, 2.5 g/g). The mitochondrial extracts (40 μg) were then resolved in a BN-PAGE gel [19,20] followed by western blotting with 4-HNE (Abcam, 1:500) and Core-2 (Abcam,#ab14745;1:1000).

### Statistical analysis

Data presented as mean ± SEM, were analyzed using Prism 6.0 (GraphPad Software Inc., San Diego, CA). The Student’s *t*-test was used to compare differences between the mean of two groups at a 95% level of confidence. P-values ≤ than 0.05 were considered statistically significant.

## Results and Discussion

### Sepsis caused an increase in lipid peroxidation

**Figure 1:**
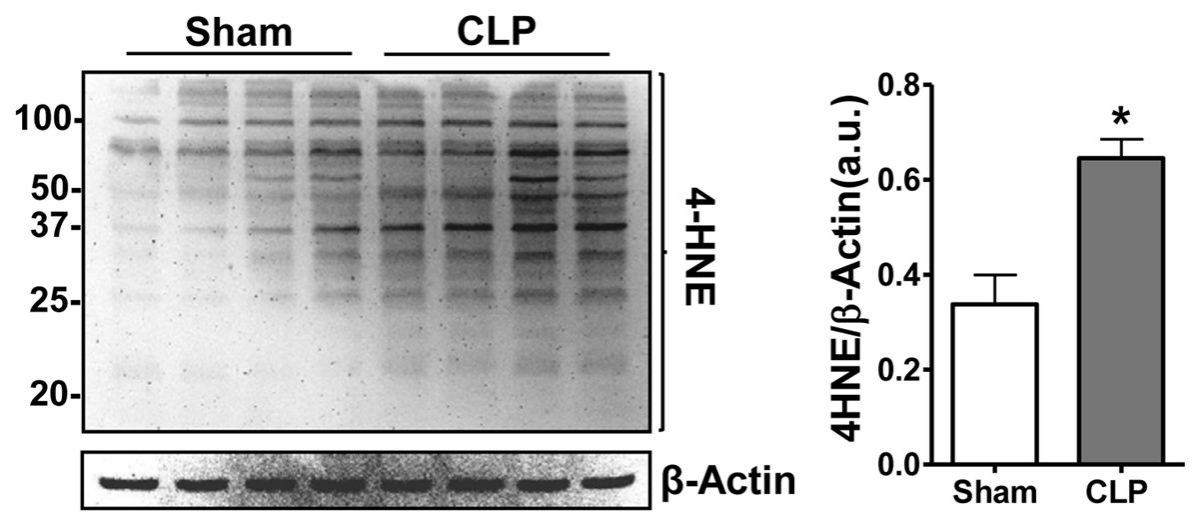
Sepsis increased renal 4-HNE protein adduction. Representative 4-HNE western blot from mice subjected to CLP (sepsis) or sham surgery. Actin was used as a loading control. Densitometry showing band intensity 4-HNE/actin. Mice subjected to CLP (18 hr) showed increased 4-HNE compared to sham animals. *P<0.05 vs. sham; n = 4/group.

We first sought to determine whether sepsis induces endogenous 4-HNE production within the kidney. A representative western blot showing 4-HNE levels in total renal homogenates in the CLP group compared to the Sham group are presented in Figure 1. Densitometry revealed significant increases in 4-HNE protein adduction in CLP compared to Sham. Actin was used as a loading control. To our knowledge, this is the first report showing increased endogenous renal 4-HNE protein adduction during sepsis. Hussain et al. showed increased 4-HNE adduction within the diaphragm after administration of lipopolysaccharide, a model of endotoxemia [21].

### Sepsis caused respiratory complex III lipid peroxidation in renal mitochondrial fractions

**Figure 2:**
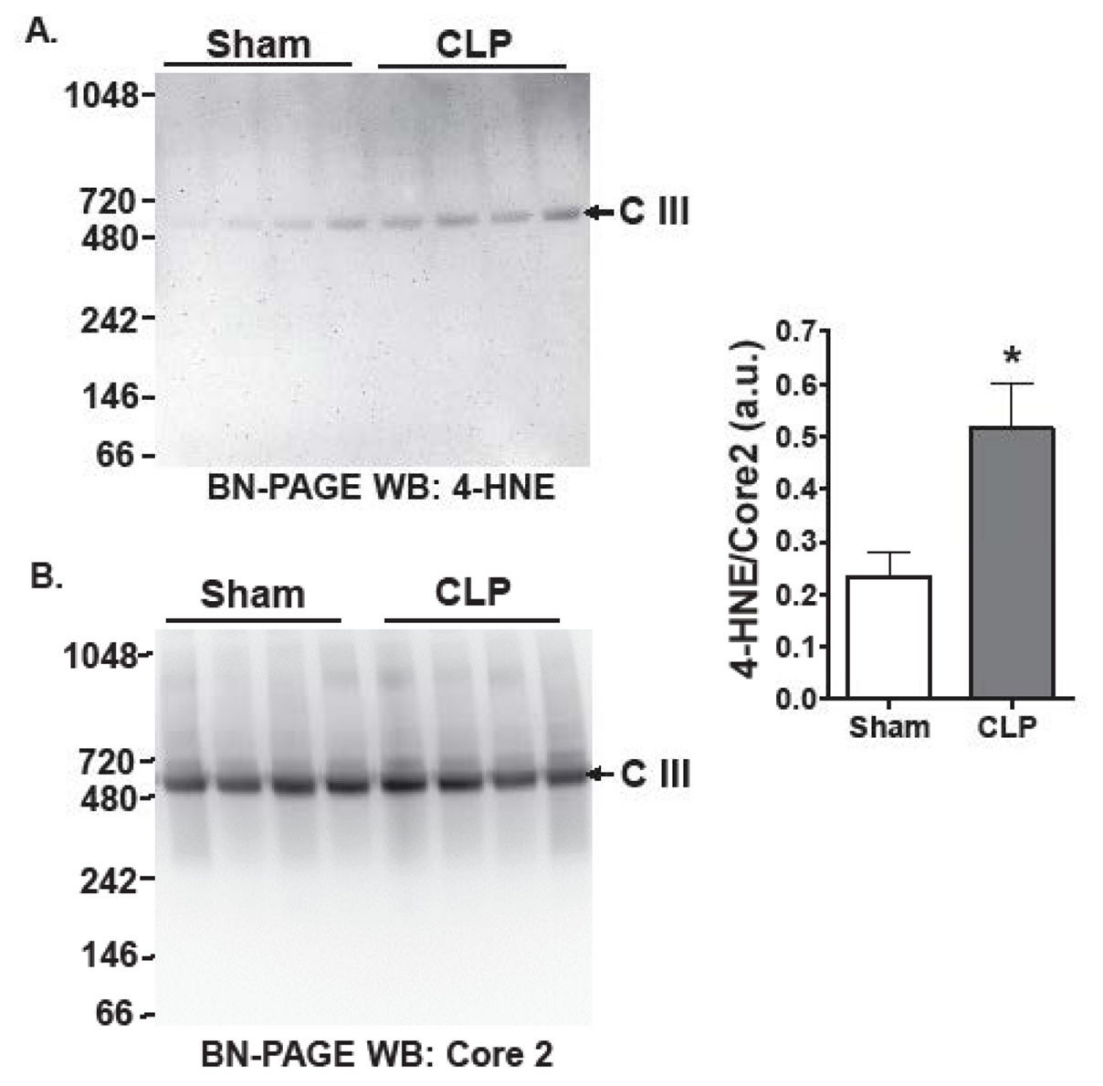
4-HNE adduction of respiratory complex III. A. Representative 4-HNE western blot (WB) of blue native gel electrophoresis (BN-PAGE) of renal mitochondria isolated from mice subjected to CLP (sepsis) or sham surgery. B. Membrane from A was stripped and probed with antibody to Core-2 (as marker of complex III), showing equal levels of respiratory complex III in sham and CLP samples. Densitometry of band intensity 4-HNE/Core2 revealed increased 4-HNE adduction of complex III compared to sham animals *P<0.05 vs. sham; n = 4/group.

Next, we wanted to determine whether lipid peroxidation was localized to the mitochondrial respiratory complexes, since our earlier studies showed that complex III activity was significantly declined at this time point (18 hr) post CLP [9]. Blue-native gel electrophoresis (BN-PAGE) was used to resolve different respiratory complexes without dissociating critical subunits [19,20]. To assess lipid peroxidation within renal mitochondrial respiratory complexes following CLP, solubilized renal mitochondrial extracts were resolved on a BN-PAGE gel followed by western blotting for 4-HNE. 4-HNE staining was increased within a single band corresponding to the molecular weight of complex III (~500 kD) in CLP, but not in Sham groups (Figure 2). The membrane was stripped and reprobed with an antibody to Core 2 (Complex III subunit) which showed equal loading of mitochondrial complex III in all samples. Densitometry revealed significant increases in 4-HNE protein adduction when compared to Core-2.

Given the broad staining pattern observed in Figure 1 using SDS-PAGE, it was surprising that complex III, which is composed of 11 subunits, was selectively targeted. Further studies are needed to determine which subunit within complex III is adduced with 4-HNE and whether adduction directly leads to complex III inactivation. Interestingly, Ullrich et al. demonstrated that addition of exogenous 4-HNE to renal mitochondria resulted in adduction to predominantly intermembrane space proteins, which is where the majority of complex III subunits are located [22]. Andrinega et al. [23] showed increased 4-HNE adduction in liver mitochondrial proteins following chronic ethanol consumption using two dimensional BN-PAGE. Another recent report showed that doxorubicin increased 4-HNE adduction of key mitochondrial proteins within cardiac tissue, but these were not located within complex III [24]. Together, these findings along with ours suggest that 4-HNE adduction within mitochondria can occur under conditions of oxidative stress and may contribute to mitochondrial injury. This also supports our recent findings that the mitochondrial antioxidant MitoTEMPO can protect mitochondrial function in the kidney during sepsis [9].

In conclusion, the key finding we present here is the first demonstration that sepsis increases renal mitochondrial lipid peroxidation and 4-HNE production, which may contribute to the mitochondrial damage we previously reported [9]. Further studies will be directed at identification of the specific mitochondrial proteins targeted for lipid peroxidation, especially within complex III since our earlier studies showed a decline in complex III activity during sepsis. Identification of such targets could provide a molecular mechanism explaining how sepsis leads to renal complex III inactivation, and more importantly to design novel therapeutic strategies designed to block lipid peroxidation and preserve mitochondrial function during sepsis.

## References

[1] Schrier RW, Wang W (2004). Acute renal failure and sepsis. N Engl J Med.

[2] Boueiz A, Hassoun PM (2009). Regulation of endothelial barrier function by reactive oxygen and nitrogen species. Microvasc Res.

[3] Brealey D, Singer M (2003). Mitochondrial Dysfunction in Sepsis. Curr Infect Dis Rep.

[4] Crouser ED (2004). Mitochondrial dysfunction in septic shock and multiple organ dysfunction syndrome. Mitochondrion.

[5] Galley HF (2011). Oxidative stress and mitochondrial dysfunction in sepsis. Br J Anaesth.

[6] Holthoff JH, Wang Z, Seely KA, Gokden N, Mayeux PR (2012). Resveratrol improves renal microcirculation, protects the tubular epithelium, and prolongs survival in a mouse model of sepsis-induced acute kidney injury. Kidney Int.

[7] Wang Z, Holthoff JH, Seely KA, Pathak E, Spencer HJ (2012). Development of oxidative stress in the peritubular capillary microenvironment mediates sepsis-induced renal microcirculatory failure and acute kidney injury. Am J Pathol.

[8] Wu L, Gokden N, Mayeux PR (2007). Evidence for the role of reactive nitrogen species in polymicrobial sepsis-induced renal peritubular capillary dysfunction and tubular injury. J Am Soc Nephrol.

[9] Patil NK, Parajuli N, MacMillan-Crow LA, Mayeux PR (2014). Inactivation of renal mitochondrial respiratory complexes and manganese superoxide dismutase during sepsis: mitochondria-targeted antioxidant mitigates injury. Am J Physiol Renal Physiol.

[10] Takasu O, Gaut JP, Watanabe E, To K, Fagley RE (2013). Mechanisms of cardiac and renal dysfunction in patients dying of sepsis. Am J Respir Crit Care Med.

[11] Esterbauer H, Schaur RJ, Zollner HO (1991). Chemistry and biochemistry of 4-hydroxynonenal, malonaldehyde and related aldehydes. Free Radic Biol Med.

[12] LoPachin RM, Gavin T, Petersen DR, Barber DS (2009). Molecular mechanisms of 4-hydroxy-2-nonenal and acrolein toxicity: nucleophilic targets and adduct formation. Chem Res Toxicol.

[13] Humphries KM, Szweda LI (1998). Selective inactivation of alpha-ketoglutarate dehydrogenase and pyruvate dehydrogenase: reaction of lipoic acid with 4-hydroxy-2-nonenal. Biochemistry.

[14] Humphries KM, Yoo Y, Szweda LI (1998). Inhibition of NADH-linked mitochondrial respiration by 4-hydroxy-2-nonenal. Biochemistry.

[15] Marine A, Krager KJ, Aykin-Burns N, MacMillan-Crow LA (2014). Peroxynitrite induced mitochondrial biogenesis following MnSOD knockdown in normal rat kidney (NRK) cells. Redox Biol.

[16] Fritz KS, Petersen DR (2013). An overview of the chemistry and biology of reactive aldehydes. Free Radic Biol Med.

[17] Rhee SG (1999). Redox signaling: hydrogen peroxide as intracellular messenger. Exp Mol Med.

[18] Munusamy S, Saba H, Mitchell T, Megyesi JK, Brock RW (2009). Alteration of renal respiratory complex-III during experimental type-1 diabetes. BMC Endocr Disord.

[19] Saba H, Batinic-Haberle I, Munusamy S, Mitchell T, Lichti C (2007). Manganese porphyrin reduces renal injury and mitochondrial damage during ischemia/reperfusion. Free Radic Biol Med.

[20] Schagger H (2001). Blue-native gels to isolate protein complexes from mitochondria. Methods Cell Biol.

[21] Hussain SN, Matar G, Barreiro E, Florian M, Divangahi M (2006). Modifications of proteins by 4-hydroxy-2-nonenal in the ventilatory muscles of rats. Am J Physiol Lung Cell Mol Physiol.

[22] Ullrich O, Grune T, Henke W, Esterbauer H, Siems WG (1994). Identification of metabolic pathways of the lipid peroxidation product 4-hydroxynonenal by mitochondria isolated from rat kidney cortex. FEBS Lett.

[23] Andringa KK, Udoh US, Landar A, Bailey SM (2014). Proteomic analysis of 4-hydroxynonenal (4-HNE) modified proteins in liver mitochondria from chronic ethanol-fed rats. Redox Biol.

[24] Zhao Y, Miriyala S, Miao L, Mitov M, Schnell D Redox proteomic identification of HNE-bound mitochondrial proteins in cardiac tissues reveals a systemic effect on energy metabolism after doxorubicin treatment. Free Radic Biol Med.

